# An Investigation of the High-Frequency Ultrasonic Vibration-Assisted Cutting of Steel Optical Moulds

**DOI:** 10.3390/mi12040460

**Published:** 2021-04-19

**Authors:** Canbin Zhang, Chifai Cheung, Benjamin Bulla, Chenyang Zhao

**Affiliations:** 1 State Key Laboratory of Ultra-Precision Machining Technology, Department of Industrial and Systems Engineering, The Hong Kong Polytechnic University, Hung Hom, Kowloon, Hong Kong, China; benny.cheung@polyu.edu.hk (C.C.); zhaochenyang@hit.edu.cn (C.Z.); 2 Son-x Gmbh, 52078 Aachen, Germany; benjamin.bulla@son-x.com; 3 School of Mechanical Engineering and Automation, Harbin Institute of Technology, Shenzhen 518055, China

**Keywords:** high frequency, ultrasonic-assisted vibration cutting, difficult-to-machine material, spherical steel mould, ultra-precision machining

## Abstract

Ultrasonic vibration-assisted cutting (UVAC) has been regarded as a promising technology to machine difficult-to-machine materials such as tungsten carbide, optical glass, and hardened steel in order to achieve superfinished surfaces. To increase vibration stability to achieve optical surface quality of a workpiece, a high-frequency ultrasonic vibration-assisted cutting system with a vibration frequency of about 104 kHz is used to machine spherical optical steel moulds. A series of experiments are conducted to investigate the effect of machining parameters on the surface roughness of the workpiece including nominal cutting speed, feed rate, tool nose radius, vibration amplitude, and cutting geometry. This research takes into account the effects of the constantly changing contact point on the tool edge with the workpiece induced by the cutting geometry when machining a spherical steel mould. The surface morphology and surface roughness at different regions on the machined mould, with slope degrees (SDs) of 0°, 5°, 10°, and 15°, were measured and analysed. The experimental results show that the arithmetic roughness $S_{a}$ of the workpiece increases gradually with increasing slope degree. By using optimised cutting parameters, a constant surface roughness $S_{a}$ of 3 nm to 4 nm at different slope degrees was achieved by the applied high-frequency UVAC technique. This study provides guidance for ultra-precision machining of steel moulds with great variation in slope degree in the pursuit of optical quality on the whole surface.

## 1. Introduction

Steel is a fascinating die material in the precision mould industry for injection moulding of plastic optical lenses and low-Tg glass. To machine steel materials, coated carbide tools [1,2] and cubic boron nitride (CBN) tools [3] are widely applied. Diamond tools possess a nanometric edge radius, form reproducibility, and excellent wear resistance, so they are extensively used in ultra-precision machining technology, such as fly cutting and single-point diamond turning (SPDT), and are capable of producing components with submicrometric form accuracy and surface roughness in the nanometre range on plastic and non-ferrous materials such as copper, aluminium, brass, etc. [4]. However, diamond machining of ferrous materials such as steel is not amenable due to excessive tool wear caused by the chemical affinity of carbon to iron [5]. Guo et al. [6] conducted a critical review of the chemical wear and wear-suppressing methods of diamond tools in the diamond cutting of ferrous materials. To reduce the chemical reaction and wear on the diamond tool and to hence improve the surface quality of the workpiece, researchers have been putting great efforts into the development of a variety of strategies. These strategies include creating a cryogenic [7] or carbon-saturated inert gas atmosphere [8], applying vibration-assisted machining [9], depositing a protective coating on the cutting tool [10], and using a ceramic tool [11].

Taking into consideration ease and economical machine setup as well as great machining stability and reliability, vibration-assisted machining has been considered a promising technique with widespread use in the industry. Vibration-assisted machining enables superior cutting performance, such as smaller cutting forces, better surface finishing, higher cutting stability, and longer tool life [12,13]. Moriwaki and Shamoto [9] first used ultrasonic vibration-assisted cutting (UVAC, Conventional Ultrasonic Vibration Cutting, CUAC, or 1D UVC) for ultra-precision diamond turning of stainless steel. A mirror-like quality with surface roughness of 26 nm was achieved, and this demonstrated the technical feasibility of ultra-precision diamond machining of steel. Subsequently, the technique of ultrasonic elliptical vibration cutting (UEVC) was firstly proposed by Shamoto and Moriwaki [12] and was used to machine hardened steel [13]. The experimental results showed that smaller cutting forces and longer tool life were achieved by the UEVC method in comparison to those in both conventional cutting and CUVC.

The optical surface quality for which the required surface roughness can be roughly determined as less than 10 nm according to various optical functions [14,15] is vital for ensuring the functional performance of the workpiece for optical application. Zhang et al. [16] conducted face-turning experiments on hardened steel by using the UEVC method with poly-crystalline diamond (PCD) tools under various nominal cutting speeds, depths of cut, and feed rates. Mirror-like surfaces were obtained, and the surface roughness of 10 nm was achieved under an optimal combination of cutting parameters. However, a constant surface roughness of less than 10 nm is still challenging in UVAC or UEVC of steel with a common vibration frequency of less than 40 kHz.

To study the influence of vibration frequency on surface roughness, Klocke et al. [17] conducted ultrasonic vibration-assisted turning under various parameters on steel with hardnesses of both 35 HRC and 50 HRC at vibration frequencies of both 40 kHz and 60 kHz. By comparing the obtained surface roughness, they found that the surface roughness at a vibration frequency of 60 kHz was more stable and smaller (most are below 10 nm). This may be due to the fact that a smaller amplitude is required for the same contact ratio between the tool and workpiece during the cutting process by increasing the vibration frequency from 40 kHz to 60 kHz, leading to a stable cutting process and thus higher surface quality. More importantly, the stable cutting process is due to the smaller cutting force resulting from a smaller upfeed stroke in each vibration cutting cycle by increasing the vibration frequency.

Based on this, ultrasonic tooling systems with operating frequencies of 80 kHz [18] and 104 kHz [19] were developed by the German company Son-x GmbH and have been used in diamond turning of spherical and aspherical surfaces on steel with sub-micrometre form accuracy and nanometric surface roughness. Unlike the conventional ultrasonic tooling system in which the sonotrode vibrates in the longitudinal mode or the first-order bending mode, the sonotrode in the 104 kHz vibration system works in a multi-order bending mode. This mode produces a primary transversal vibration in the cutting direction as well as a secondary longitudinal vibration in the thrust direction, both of which are fused into a new vibration trajectory [19].

Although sub-micrometre form accuracy and nanometric surface roughness can be achieved with the newly developed 104 kHz ultrasonic tooling system, there are few studies that systematically investigate the effect of cutting parameters on surface roughness with this vibration setup so the setup’s machining stability and reliability need to be further examined. Moreover, little research on the surface finish in ultrasonic vibration-assisted cutting of steel considers the effects of the machining geometry of the workpiece. Therefore, this study aimed to investigate the effect of cutting parameters such as nominal cutting speed, feed rate, tool nose radius, and vibration amplitude on the surface roughness of the workpiece by making use of the Taguchi orthogonal experiment. In addition, the surface roughness of spherical moulds with various slope degrees were measured and analysed to explore the effect of the machined geometry on surface quality in the ultrasonic vibration-assisted cutting of spherical optical steel moulds.

## 2. Working Principle and Cutting Mechanics Of UVAC

### 2.1. Cutting Mechanics

In ultrasonic vibration-assisted cutting, the cutting tool is equipped with vibration moving up and down at an ultrasonic frequency, parallel to the cutting direction. The vibration displacement and the vibration speed can be determined by Equations (1) and (2), respectively [20]:

(1) x=Asin(2πft)

(2) $$x^{\prime }=2\pi fAcos\left(2\pi ft\right)$$

where *A* and *f* are the vibration amplitude and vibration frequency, respectively. If the maximal speed of vibration (2πfA) is larger than the nominal cutting speed $v_{c}$, the separation between the rake face of the cutting tool and the cutting zone of the workpiece occurs. This separation is a characteristic of ultrasonic vibration-assisted cutting that can enhance lubrication and cooling conditions and thus reduce the cutting forces and tool wear, thereby improving the cutting stability and the surface finish [12,13].

**Figure 1:**
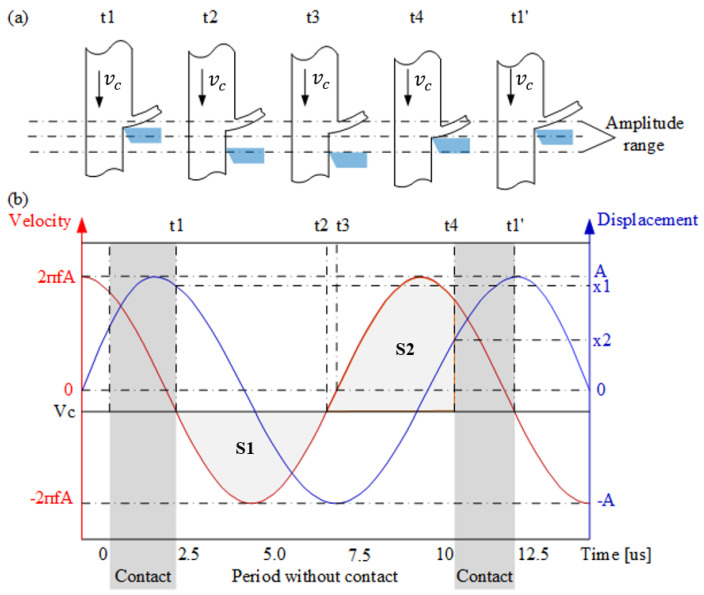
(**a**) Schematic diagram of the position relationship between the tool and the workpiece in UVAC. (**b**) The kinematics of UVAC.

To better understand the cutting process of UVAC, Figure 1a is a schematic diagram of the relative position between the tool tip and the workpiece in the UVAC process. Several important time moments at *t*1, *t*2, *t*3, *t*4, and *t*1’ in one cutting cycle are discussed and their corresponding dynamics are shown in Figure 1b. The three curves represent the vibration displacement, vibration speed, and nominal cutting speed relative to the static coordinate system. At the moments of *t*1 and *t*2, the vibration speed and the nominal cutting speed are equal. *t*1 and *t*2 can be determined by solving Equation (3) as follows [20]:

(3) $$2\pi fAcos\left(2\pi ft\right)=-v_{c}$$

At *t*1, the cutting tool starts to separate from the cutting zone of the workpiece, and the corresponding vibration displacement is $x_{2}=Asin\left(2\pi ft1\right)$. At *t*2, the distance between the tool and the workpiece reaches the maximum value, which is the area of S1, as shown in Figure 1b. After t2, the distance between the tool and the workprice starts to decrease. At *t*3, the vibration motion reaches the lowest point, and then starts to move upward. The area of S2 as shown in Figure 1b shows a decreasing distance between the tool and the cutting zone of the workpiece after *t*2. When the areas of these two parts are equal (S1 = S2), at *t*4, the tool contacts the cutting zone of the workpiece again for cutting until *t*1’, and then one cutting cycle T is completed. After this, the tool and the cutting zone of the workpiece separate again and enter the next cutting cycle. The vibration position at *t*4 is $x_{2}=Asin\left(2\pi ft4\right)$.

From *t*1 to *t*4, the displacement of vibration is Δx:

(4) $$\Delta x=x_{1}-x_{2}=Asin(\left(2\pi ft1\right)-Asin\left(2\pi ft4\right)$$

The displacement of the workpiece is $s=v_{c}\left(t4-t1\right)$. The condition for the tool contacting the cutting zone of the workpiece again is Δx=s:

(5) $$\Delta x=s\to Asin\left(2\pi ft1\right)-Asin\left(2\pi ft4\right)=v_{c}\left(t4-t1\right)$$

The time point *t*4 can be solved numerically. To be specific, it can be solved by gradually increasing the number of time units dt, namely *n*, starting from *t*1. When $Asin\left(2\pi ft1\right)-Asin\left(2\pi f\left(t1+ndt\right)\right)=v_{c}ndt$ is satisfied within the error tolerance, it is assumed that n=N. Therefore, t4=t1+Ndt.

The contact ratio of the tool tip with the cutting zone of the workpiece, namely ‘Duty Cycle (*DC*)’, is expressed as follows [20]:

(6) $$DC=\frac{T-(t4-t1)}{T}=\left(T-Ndt\right)f$$

**Figure 2:**
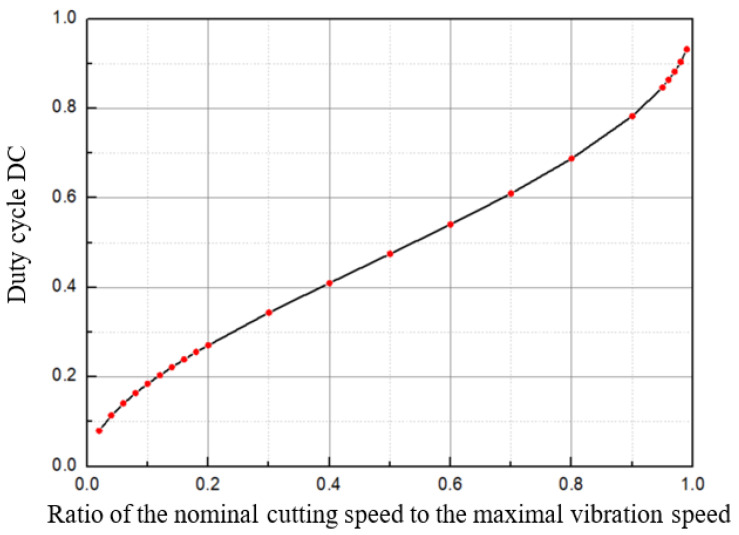
Relationship between the duty cycle and the speed ratio.

Given that the vibration frequency *f* is constant, the speed ratio of nominal cutting speed $v_{c}$ to the maximal vibration speed 2πfA can be varied by changing the value of $v_{c}$ or *A*. When the speed ratio is ascertained, the corresponding duty cycle can be determined by solving Equations (3)–(6). In this way, the duty cycle for various speed ratios can be calculated. Figure 2 shows the duty cycle as a function of the speed ratio of the nominal cutting speed to the maximal vibration speed. The duty cycle decreases with decreasing speed ratio. It is believed that a smaller *DC* means better lubrication and cooling, which leads to a smaller cutting force and better surface quality [21]. In UVAC, under constant nominal cutting speed $v_{c}$, a higher vibration frequency leads to a smaller speed ratio and thus a smaller *DC* at a constant vibration amplitude or a smaller vibration amplitude at a constant *DC*. Both result in stabilised vibration and better surface quality.

### 2.2. The Applied High-Frequency UVAC

**Figure 3:**
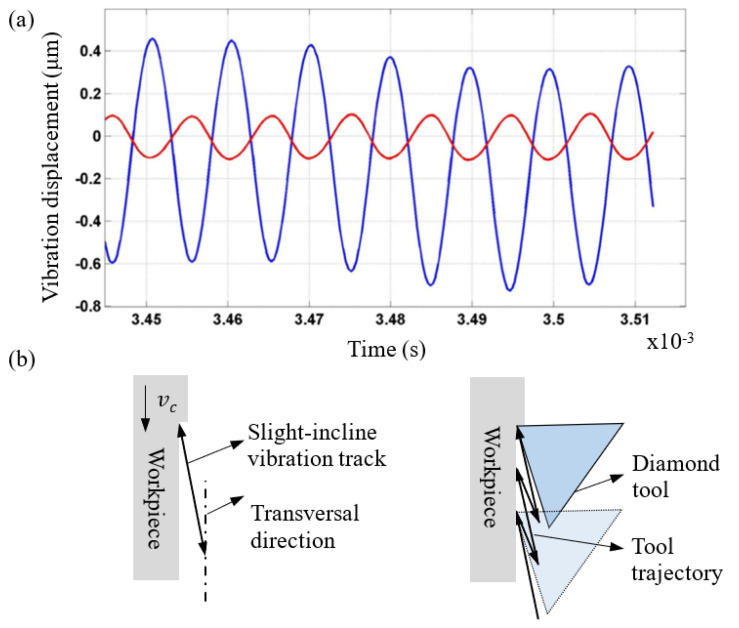
(**a**) Diamond tool tip transversal vibration displacement (blue line) and longitudinal vibration displacement (red line) during the cutting stage [19]. (**b**) Schematic of the tool tip movement of the slight-incline UVAC.

The vibration equipment Son-X UTS2 was used in this research. This ultrasonic tooling system was designed and described in the previous literature [19]. The transducer and sonotrode are actuated at the resonant frequency of 104 kHz, and the sonotrode works in the multi-order bending mode. During the cutting stage, the tool tip has both transversal vibration (blue line) and longitudinal vibration (red line), with a phase difference of 180°, as shown in Figure 3a. The amplitude ratio of the transversal and longitudinal vibration is about 5:1 (i.e., the longitudinal vibration amplitude is 200 nm when the transversal vibration amplitude is 1 μm). The differences in phase and amplitude between transversal and longitudinal vibration are finally fused into a slight-incline vibration track relative to the transversal direction, namely the cutting direction, and this vibration leads to a saw-like trajectory of the tool tip relative to the workpiece under a nominal cutting speed $v_{c}$, as shown in Figure 3b. Compared with UVAC, it is interesting to note that the tool tip includes an additional component motion in the thrust direction. This enables the tool tip to completely separate from the finished surface when the tool is withdrawn from the workpiece. This characteristic can facilitate contact between the coolant and lubricant, and the flank face of the cutting tool, or contact between oxygen in the air and the freshly cutting surface when cutting ferrous materials [22,23], thereby reducing the tool wear rate.

## 3. Experiment

### 3.1. Machining Dimension and Material of the Moulds

**Figure 4:**
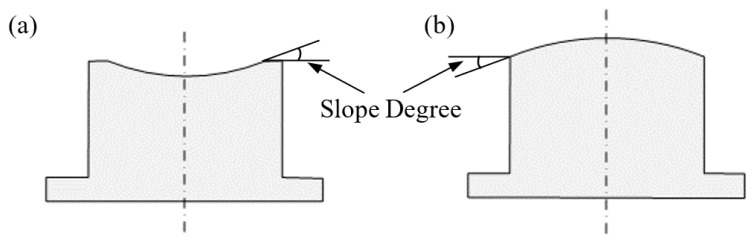
Diagram of the spherical moulds: (**a**) concave and (**b**) convex.

Figure 4a,b show diagrams of a concave and convex spherical mould, respectively. Both have a spherical surface with a radius of 15 mm. The concave mould has an aperture of 12 mm and a maximum slope degree of 23.6°. The convex mould has an aperture of 15 mm and a maximum slope degree of 30°.

**Figure 5:**
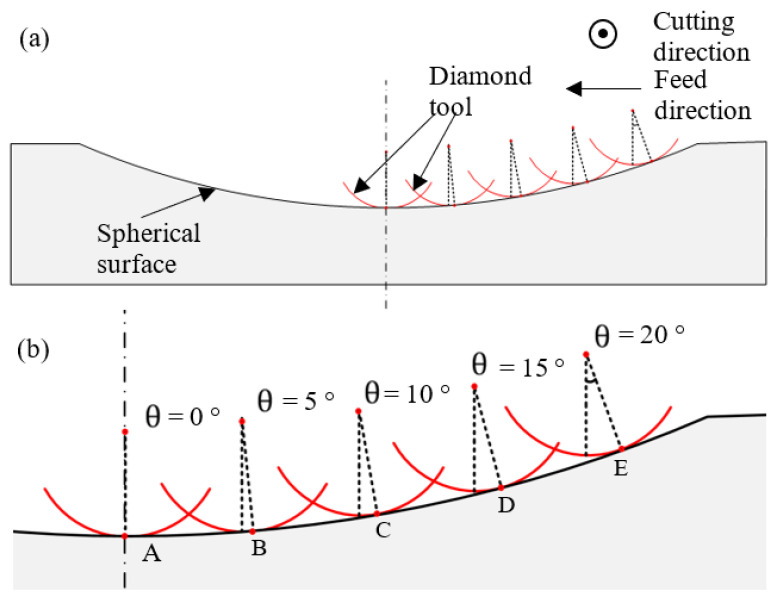
(**a**) Schematic diagram of cutting a concave spherical mould. (**b**) A partial enlarged view of (**a**).

The geometry of the spherical surface could have an effect on the cutting force components as well as the surface roughness of the machined workpiece since it leads to variation in the cutting position of the tool edge. Taking the cutting process of the concave mould as shown in Figure 5, during the cutting process, the cutting edge of the diamond tool moves inwards to the center of the workpiece along the spherical surface. In this process, the point on the workpiece in contact with the cutting tool transfers from E to D, C, and B, and then to A, which has a slope degree of 20°, 15°, 10°, 5°, and 0°, respectively. This process also results in variation in the cutting point on the tool and their cutting angles θ, namely the angle between the symmetric line of the tool rake face and the normal line of the tool edge at these points, which are correspondingly 20°, 15°, 10°, 5°, and 0°. The variation in the cutting position and the cutting angle could possibly induce a difference in the cutting force and surface quality, which is worth studying.

The material used in this study was Mirrax 40 steel. Mirrax 40 is a remelted stainless steel prehardened to 40 HRC. It has excellent machinability, polishability, ductility, and toughness, which make it appropriate for injection moulds, extrusion dies, and flow moulds. The chemical composition of Mirrax 40 steel includes (wt.%): C: 0.21, Si: 0.9, Mn: 0.45, Cr: 13.5, Mo: 0.2, Ni: 0.6, and V: 0.25.

### 3.2. Procedures and Setup

**Figure 6:**
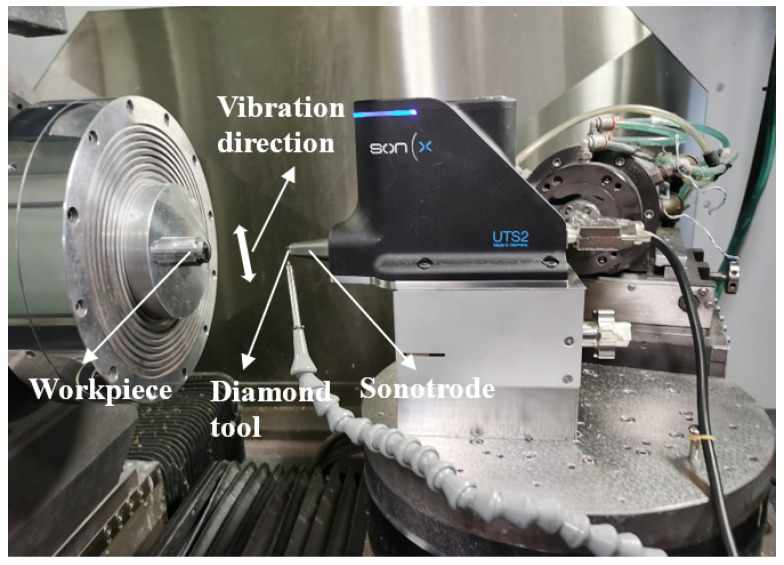
Photo of the machining setup.

The spherical surface turning experiments were conducted on an ultra-precision machine (Nanotech 450 from Nanotechnology Inc., Los Angeles, CA, USA) equipped with an ultrasonic vibration-assisted diamond machining system (i.e., Son-X UTS2 from Son-X GmbH Germany), which was installed on the *Z*-axis guide, as shown in Figure 6. In each experiment, the constant surface speed (CSS) mode was utilised. The spindle rotational speed increased steadily to keep the nominal cutting speed constant at different areas on the spherical surface with various diameters when the cutting tool moved inwards. Since the spindle rotational speed could not increase infinitely with the cutting tool moving close to the centre of the workpiece, it was kept constant until the cut was finished after the spindle speed reached 300 rpm. A rough cut of 10 μm per rev, 5 μm depth-of-cut, and 4 m/min cutting speed were firstly conducted to eliminate the dimensional error of the workpiece, followed by a finishing cut under specific parameters. After the finishing cut, the surface quality of the machined mould was evaluated and the surface roughness was measured using the New Zygo NexView Optical Profiler (USA). To directly and accurately measure the surface roughness of the areas at different slope degrees on the mould surface, an angle inclinometer was used for rotation of the workpiece. The arithmetic roughness $S_{a}$ of the areas with the slope degrees of 0°, 5°, 10°, and 15° were measured and analysed.

### 3.3. Experimental Design Using the Taguchi Method

The Taguchi method [2,3,24] is an engineering approach used to investigate the effects of process parameters on the target performance for optimisation of these parameters. Designing an orthogonal array test can greatly reduce the experimental trials and then quickly identify key parameters. The possible key parameters in ultrasonic vibration-assisted cutting that may have significant effects on surface roughness are the tool radius (TR), cutting speed (CS, namely nominal cutting speed), vibration amplitude, feed rate (FR), and surface form. For the Son-X UTS2, the vibration amplitude of the vibrator is associated with the input current (IC). The larger the input current, the larger the vibration amplitude. As a result, the five parameters including TR, CS, IC, FR, and surface form were identified as five experimental factors for ultrasonic vibration-assisted cutting of the spherical moulds in this study. Based on the previous study, three levels for each factor (except the surface form with two levels of concave and convex) were selected to cover the research interest, as shown in Table 1. The rake angle and clearance angle of the cutting tool were 0° and 10°. The peak-to-peak vibration amplitudes were approximately 1.2, 1.8, and 2.4 μm in response to the input currents of 20, 30, and 40 mA, respectively.

### 3.4. Data Analysis for the Taguchi Method

The target optimisation can be categorised into nominal-the-best type, the-smaller-the-better type, the-larger-the-better type, etc. Generally, the signal-to-noise ratio (S/N) is used as the objective function for optimising the process parameters. It should be noted that the equation for S/N could vary from one optimisation type to another. As the surface roughness of the spherical surface cutting process is a the-smaller-the-better type problem, S/N is defined by Equation (7) [24] as follows:

(7) $$S/N=-10log_{10}\left[\frac{1}{n}\sum_{i=1}^{n}y_{i}^{2}\right]$$

where $y_{i}$ is the value of the target characteristic under different noise conditions and *n* is the number of noise conditions.

In this experiment, $y_{i}$ is the arithmetic roughness $S_{a}$ of different areas on the mould surface, which are characterised by the slope degree, and n is the number of the measured slope degrees. To optimise the parameters for spherical surface cutting, levels that maximise *S*/*N* should be selected for the factors that have a significant effect on surface roughness.

## 4. Results and Discussions

Table 2 summarises the measured arithmetic roughness $S_{a}$ at the four slope degrees on the machined spherical moulds, S/N, and $\bar{S_{a}}$ (the mean of $S_{a}$) for each $L_{18}$ orthogonal array experiment. By comparing these values, the effect of the levels for each factor on surface roughness can be identified and the optimal level for each factor can be determined.

### 4.1. Surface Roughness Analysis

#### 4.1.1. Average Arithmetic Roughness $\bar{S_{a}}$ for Various Levels of Each Factor

**Figure 7:**
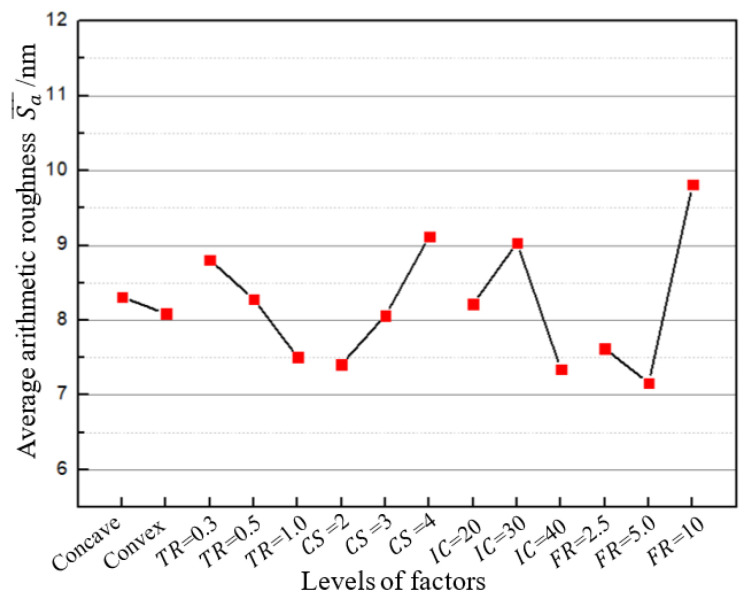
Average arithmetic roughness $\bar{S_{a}}$ of four slope degrees under various levels for each factor.

Figure 7 shows the average arithmetic roughness $\bar{S_{a}}$ for various levels of each factor. For the level of 0.3 mm of the tool radius, its value is determined as the mean value of $\bar{S_{a}}$ with the same tool radius. (i.e., (6.7 + 11.8 + 7.2 + 8.2 + 12.6 + 6.4) nm/6 = 8.8 nm). It is found that the feed rate has a great effect on the arithmetic roughness, while surface form has the least, which could be negligible compared with other factors. Tool radius, cutting speed, and input current have almost the same difference in arithmetic roughness under the selected levels. In view of the effect of the level for each factor, cutting speed and feed rate display upward and down-then-upward trends, respectively. This finding is consistent with previous study [16]. A larger cutting speed causes less overlapping cutting cycles and thus a larger upfeed stroke in each vibration cycle, requiring a relatively higher material load to be removed. Moreover, a larger cutting speed leads to a higher ratio of the cutting speed to the maximal vibration speed, increasing the contact time between the tool and the workpiece, which reduces the lubrication performance and heat transfer during the cutting process. Both can induce a larger cutting force and hence poorer surface quality. Regarding the feed rate, it appears that a high feed rate results in a high material removal load and cutting force, causing instability and thus poor surface quality in the cutting process. By contrast, a too low feed rate may cause the uncut chip thickness to become comparative to the radius of the tool edge, which can induce significant ploughing and rubbing and thus lead to poor surface quality [16].

The tool radius exhibits a peculiar and interesting downward trend of arithmetic roughness with increasing level value. It is noted that the thrust force increases with increasing tool radius under the same cutting conditions. Nath et al. [25] found that a 0.6-mm tool radius achieved better surface quality than a smaller or a larger one when machining tungsten carbide using ultrasonic elliptical vibration cutting with a vibration frequency of 40 kHz. It was found that worse surface quality for a larger tool radius (0.8 mm) was caused by the increasing cutting force, leading to a break in the regenerative chatter suppression dynamics [26] and thus instability of the cutting process. This means that the tool radius can be improved without causing cutting instability to achieve smaller theoretical surface roughness if the vibration-assisted cutting technique can produce a smaller average force under the same cutting conditions. As shown in Figure 7, the optimal tool radius for minimum surface roughness in the applied 104 kHz UVAC technique increases to equal to or maybe larger than 1 mm. This appears to indicate that this technique may have a better cutting performance for surface finishes in the machining of steel. Moreover, the results show that all of the arithmetic roughness values under various levels of each factor are below 10 nm, which could be regarded as another justification for the superiority of the surface quality in this high-frequency ultrasonic vibration-assisted cutting technique.

Input currents exhibit an up-then-downward trend of arithmetic roughness with increasing level value. It is understandable that the surface quality can be improved by increasing the input current of the vibrator, which generates increasing vibration amplitude. Increasing the vibration amplitude leads to an increase in the maximal vibration speed and thus a decrease in the speed ratio and cutting duty cycle. This results in better lubrication as well as heat transfer and hence better surface quality. However, it seems peculiar that the surface roughness with a current of 20 mA is lower than that for 30 mA. One possible reason is that a smaller vibration amplitude makes the vibrator have better stiffness and stability so it undergoes less deflection when subjected to certain loading.

#### 4.1.2. Arithmetic Roughness $S_{a}$ for Each SD

**Figure 8:**
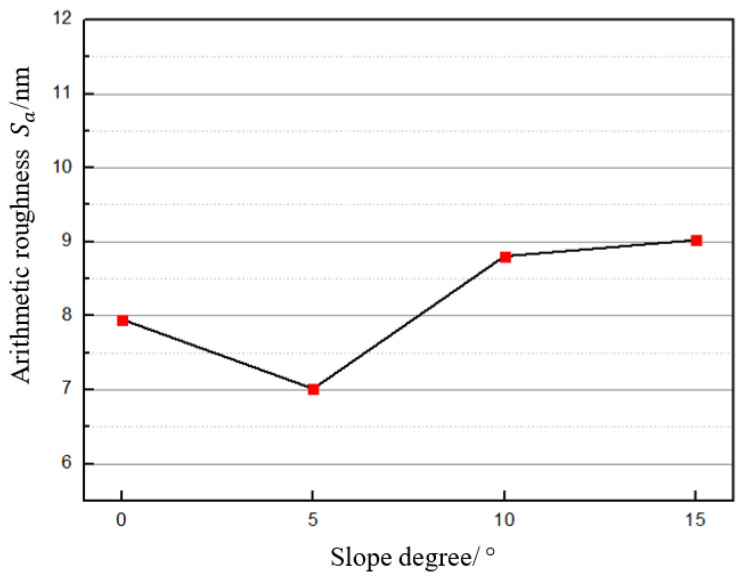
Arithmetic roughness $S_{a}$ with respect to various slope degrees.

To study the effect of various slope degrees on the surface roughness, Figure 8 summarises the arithmetic roughness $S_{a}$ as a function of the slope degree of the workpiece. For the slope degrees 0°, 5°, 10°, and 15°, their values are determined as the mean value of $S_{a}$ in all of the orthogonal array experiments at the same slope degrees of 0°, 5°, 10°, and 15°, respectively. It was found that the surface roughness exhibits an upward trend with increasing slope degree, except at the slope degree of 0°. The arithmetic roughness at 0° is higher than that for 5°. One possible reason for this is that the nominal cutting speed keeps changing with decreasing the cutting diameter in the central area of the workpiece because the rotational speed of the spindle remains constant after reaching 300 rpm in this area. This constant change in cutting speed could result in instability of the cutting process, reducing the surface quality. Another reason is that the surface morphology in this area is more likely to be scratched and influenced by the flow chips. Excluding this special area, the arithmetic roughness of other slope degrees exhibited an upward trend. This machining phenomenon is not preferable to produce a homogeneous surface finish on the surface with geometry of great difference in the slope degree so the underlying reasons for this need to be analysed for the purpose of suppression.

An attempt was made to determine the possible reasons for this phenomenon. One could be due to the variation in tool force under various tool cutting angles due to varying slope degrees of the workpiece. With the increase in slope degree of the workpiece during the cutting process, the cutting point on the tool edge changes and thus the cutting angle between the symmetric line of the tool rake face and the normal line of the tool edge at the cutting point increases as mentioned in Section 3.1. This leads to an increase in the lateral force loading on the cutting tool, which could probably cause cutting instability due to the relatively weak resistance to loading of the vibrator in the lateral direction, thus resulting in deterioration of the surface quality. To verify this speculation, tool force components during the cutting process at various cutting angles need to be measured and analysed.

**Figure 9:**
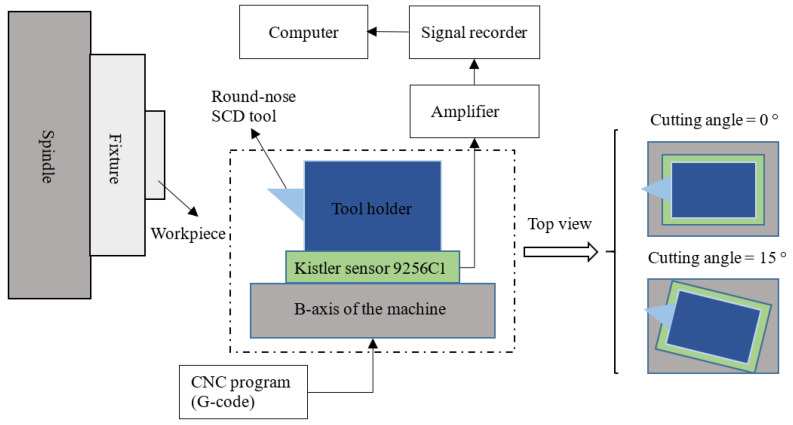
Schematic of the experimental setup for the low-frequency UVAC.

**Figure 10:**
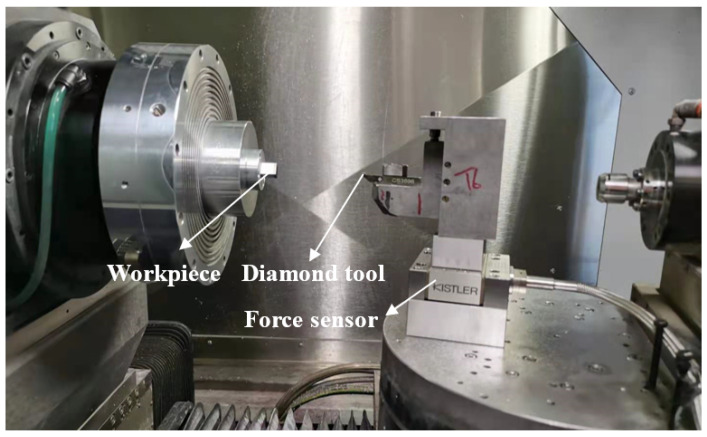
Photo of the machining setup for the low-frequency UVAC.

To investigate the effect of cutting angle on the tool force during the cutting process, the tool force at the cutting angles of 0° and 15° was measured and compared. Figure 9 and Figure 10 show a schematic and a photo of the experimental setup. The cutting tool with a nose radius of 1 mm and a rake angle of 0° was used. In the case of the cutting angle of 0°, the tool was installed with the symmetric line of the tool rake face parallel to the *Z*-axis of the machine. In contrast, in the case of the cutting angle of 15°, the cutting tool together with the force sensor were clockwise rotated along the *Y*-axis by 15° based on the condition of 0° by the B-axis of the machine (Top view). The desired nominal cutting speed, depth of cut, and feed rate of the practical cutting condition were 3 m/min, 5 μm, and 10 μm/rev, respectively. It is noted that it is impossible to acquire the actual transient tool force in the UVAC process as the vibration frequency is much larger than the natural frequency of the force sensor. As a result, the UVAC process was imitated by programming its tool trajectory as a conventional UVAC at a low frequency [27]. The imitated vibration frequency was set as 0.5 Hz with a peak-to-peak amplitude of 2 μm, and the actual nominal cutting speed was set as 14.4 μm/min to keep the upfeed stroke in each vibration cycle identical to that in the practical UVAC process.

**Figure 11:**
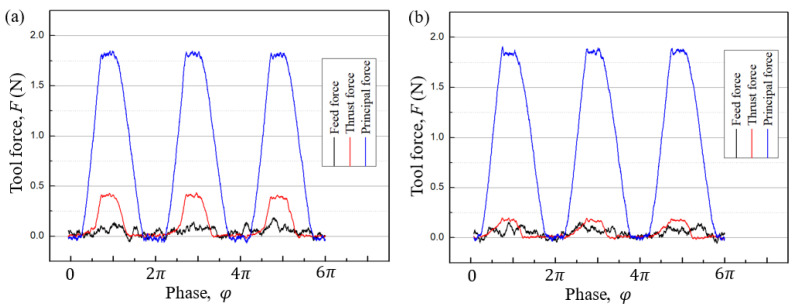
Tool force measured in the programmed grooving tests at various cutting angles: (**a**) 0° and (**b**) 15°, *f* = 0.5 Hz, *A* = 1 μm, $v_{c}$ = 14.4 μm/min, depth of cut = 5 μm, and feed rate = 10 μm/rev.

In the experiment, face turning was conducted to flatten the workpiece. Following this, the cutting tool moved towards the workpiece at the given diameter by *Z*-axis until the desired depth of cut and started cutting for an rotation angle of 90° to make the cutting process stable. After this, a programmed grooving test was conducted in the *C*-axis mode and the tool force was acquired by the Kistler force senser 9256C1. According to Arcona’s force model [28], the tool force is proportional to the material hardness of the workpiece. To avoid excessive tool wear in low-frequency vibration cutting of steel while achieving a tool force identical to that in the machining of steel, electroless plated nickel with a hardness close to that of steel was selected as the cutting material. The measured tool force at the cutting angles of 0° and 15° in the grooving tests with 3 vibration cycles is presented in Figure 11a,b, respectively.

With an increase in the cutting angle from 0° to 15°, the principal force remains constant, the thrust force decreases observably while the feed force increases slightly. Although this changing trend of tool force is consistent with our previous conjecturing, the thrust force and especially the feed force in the grooving test are within a small range (<0.5 N). The tool force in the turning machining process in which there is a smaller material removal rate is even smaller compared with the grooving test. As a result, the variation in tool force for the turning machining due to increasing cutting angle may have little influence on the cutting stability and therefore should not be regarded as the primary factor for declining surface quality at high cutting angles.

**Figure 12:**
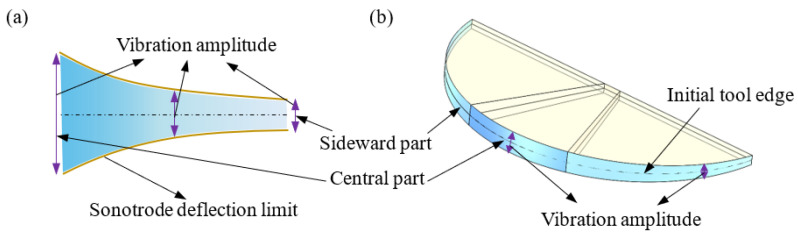
Schematic diagram of (**a**) the sonotrode deflection and (**b**) tool vibration amplitude distribution along the tool edge in the bending vibration mode.

Another possible reason could be the variation in vibration amplitude along the tool edge. As the applied ultrasonic tooling system works in the multi-order bending mode, the sonotrode undergoes bending deflection during the vibrating process, as presented in Figure 12a. Consequently, the tool tip vibrates with the largest amplitude while the vibration amplitude decreases gradually from the centre of the tool edge to both of its sides, as shown in Figure 12b. The largest vibration amplitude at the central part of the tool edge allows for better machining conditions for lubrication and heat transfer to achieve a better surface finish. During UVAC of spherical steel moulds, the central part of the tool is used to machine the geometry with a small slope degree while the sideward part is used to machine the geometry with a large slope degree. As a result, the surface roughness of the machined spherical steel mould increases with increasing slope degree.

### 4.2. Optimisation of the Level for Each Factor

**Figure 13:**
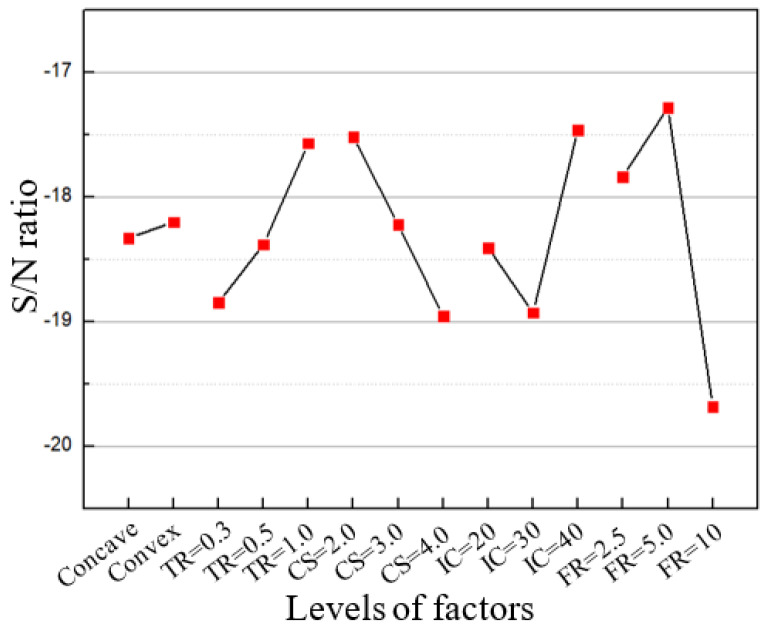
S/N ratio of levels for each factor.

**Figure 14:**
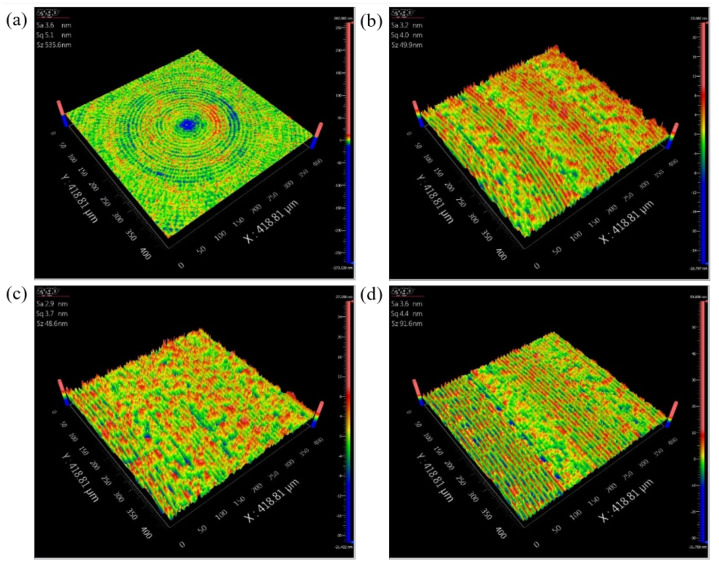
Surface morphology and measured roughness of the convex mould at different slope degrees under optimised parameters: (**a**) 0°, (**b**) 5°, (**c**) 10°, and (**d**) 15°.

To minimise the arithmetic roughness value of the machined spherical mould, the level for each factor that can maximise the S/N ratio should be selected. Figure 13 plots the S/N ratio of all levels for each factor. In practice, long-time use of UTS2 at the current of 40 mA possibly causes cutting instability, so the current of 20 mA is considered a better parameter value. As a result, the optimal combination of the level for each factor is determined to be 1 mm for tool radius, 2 m/min for cutting speed, 20 mA for input current, and 5 μm per rev for feed rate. Consequently, the convex mould was machined at the optimised parameters. Figure 14 shows the surface morphology and measured roughness of the machined convex mould at various slope degrees under optimised parameters. The arithmetic roughness $S_{a}$ of 3–4 nm could be achieved for all slope degrees. Furthermore, the arithmetic roughness $S_{a}$ is so small that the effect of the slope degree becomes insignificant. This means that the unevenness of the surface finish in machining the geometry with a great difference in slope degree using this ultrasonic tooling system can be alleviated by using an optimal combination of cutting parameters.

## 5. Conclusions

This study investigated the effect of cutting parameters as well as the geometry of a workpiece on the surface roughness using high-frequency ultrasonic vibration-assisted cutting. The ultra-precision machined spherical moulds in this study were made of steel. Some findings are given as follows:

1. Feed rate has the greatest effect on the surface roughness of the workpiece: 5 μm/res was able to achieve better surface roughness compared with 10 μm/res and 2.5 μm/res. Under the selected levels of the other factors, surface roughness decreased with increasing tool radius while it increased with increasing nominal cutting speed. The effect of input current on surface roughness remains unclear based on the obtained results.
2. To achieve a better surface finish, the optimal combination of the level for each cutting parameter is 1 mm for tool radius, 2 m/min for cutting speed, 20 mA for input current, and 5 μm per rev for feed rate.
3. Arithmetic roughness on the machined spherical steel mould exhibited a slight upward trend with an increase in slope degree from 5° to 15°, and the variation in vibration amplitude distribution along the tool edge arising in the bending vibration mode is speculated to be the possible reason.
4. Under optimal cutting conditions, the optical surface quality, with an arithmetic roughness $S_{a}$ of 3–4 nm, can be achieved at various slope Degrees. Furthermore, the unevenness of surface finishes at various slope degrees was alleviated in this ultrasonic tooling system with a combination of optimal parameters.

The results demonstrate the technical merits of using high-frequency ultrasonic vibration-assisted cutting in the machining of steel material with a superior surface finish. Further research will be conducted to continuously examine and verify the reason for the unevenness of surface quality in the machining of workpieces with complex geometries where there is a great difference in the slope degree to identify a strategy to improve the vibration setup. 

## Figures and Tables

**Table 1:** The experimental factors and their levels.

Factor	Level 1	Level 2	Level 3
A. Tool Radius TR (mm)	0.3	0.5	1.0
B. Cutting Speed CS (m/min)	2	3	4
C. Input Current IC (mA)	20	30	40
D. Feed Rate FR (μm/rev)	2.5	5	10
E. Surface form	Concave	Convex	

**Table 2:** Surface roughness of the machined spherical moulds.

Test No.	TR	CS	IC	FR	Surface Form	$S_{a}$(nm) SD = 0°	$S_{a}$(nm) SD = 5°	$S_{a}$(nm) SD = 10°	$S_{a}$(nm) SD = 15°	S/N	$\bar{S_{a}}$ (nm)
1	0.3	2	20	2.5	Convex	5.3	5.3	10.9	5.4	−17.1	6.7
2	0.3	4	30	10	Convex	12.5	11.0	12.1	11.5	−21.4	11.8
3	0.3	3	40	5	Convex	12.4	5.4	5.5	5.5	−17.9	7.2
4	0.3	2	20	2.5	Concave	6.1	6.4	9.5	10.9	−18.6	8.2
5	0.3	4	30	10	Concave	13.3	11.6	12.9	12.5	−22.0	12.6
6	0.3	3	40	5	Concave	8.1	5.4	6.1	5.8	−16.2	6.4
7	0.5	4	40	2.5	Concave	7.3	5.9	7.3	7.7	−17.0	7.0
8	0.5	2	30	5	Concave	5.7	5.9	6.5	7.4	−16.1	6.3
9	0.5	3	20	10	Concave	8.0	8.4	9.7	10.5	−19.3	9.1
10	0.5	4	40	2.5	Convex	6.8	5.9	9.8	11.6	−18.9	8.5
11	0.5	2	30	5	Convex	6.9	6.0	9.3	10.7	−18.5	8.2
12	0.5	3	20	10	Convex	13.9	8.6	9.5	9.7	−20.5	10.4
13	1	2	40	10	Convex	6.5	6.1	6.9	7.1	−16.5	6.7
14	1	3	30	2.5	Convex	5.2	6.0	7.2	7.5	−16.3	6.5
15	1	4	20	5	Convex	5.6	6.0	7.9	7.5	−16.7	6.8
16	1	2	40	10	Concave	8.0	8.3	8.4	8.5	−18.4	8.3
17	1	3	30	2.5	Concave	5.8	7.2	9.8	12.2	−19.2	8.8
18	1	4	20	5	Concave	5.7	6.9	9.4	10.3	−18.3	8.1

## Nomenclature

The following abbreviations or physical quantities are used in this manuscript:

UVAC	Ultrasonic vibration-assisted cutting
SD	Slope degree
$S_{a}$	Arithmetic surface roughness
$\bar{S_{a}}$	Average arithmetic surface roughness
CBN	Cubic boron nitride
SPDT	Single point diamond turning
CUAC or 1D UVC	Conventional ultrasonic vibration cutting
UEVC	Ultrasonic elliptical vibration cutting
PCD	Poly-crystalline diamond
*f*	Vibration frequency
*A*	Vibration amplitude
$v_{c}$	Nominal cutting speed
DC	Duty cycle
TR	Tool radius
CS	Cutting speed, namely nominal cutting speed
IC	Input current
FR	Feed rate
S/N	Signal-to-noise ratio
CSS	Constant surface speed
